# Clinical features of intracardiac thrombotic complication in patients with severe *Mycoplasma pneumoniae* pneumonia

**DOI:** 10.1186/s13052-025-01890-3

**Published:** 2025-02-11

**Authors:** Xiao Li, Bo Zhai, Yu Tang, Lei Zhang, Jing Wang, Chunna Xu, Lili Dong, Yanqiong Wang, Yanyan Su, Zhen Dong, Haiming Yang, Yuelin Shen

**Affiliations:** 1 Respiratory Department, Children’s Hospital Affiliated to Zhengzhou University, Henan Children’s Hospital, Zhengzhou Children’s Hospital, Zhengzhou, 450018 China; 2 Respiratory Department II, National Clinical Research Center for Respiratory Diseases, Beijing Children’s Hospital, Capital Medical University, National Center for Children’s Health, Beijing, 100045 China; 3 Cardiac Surgery Department, Children’s Hospital Affiliated to Zhengzhou University, Henan Children’s Hospital, Zhengzhou Children’s Hospital, Zhengzhou, 450018 China

**Keywords:** Children, Intracardiac thrombus, *Mycoplasma pneumoniae* pneumonia, Thrombosis, Thrombectomy

## Abstract

**Background:**

Intracardiac thrombus (ICT) is the rarest yet most severe complication of severe *Mycoplasma pneumoniae* pneumonia (SMPP) in children. This study aims to elucidate the clinical characteristics of patients with SMPP-related ICT.

**Methods:**

We retrospectively enrolled 68 children with SMPP (18 cases of ICT, 50 cases of non-ICT) who were admitted from January 2014 to January 2024. We compared their demographic data, clinical symptoms, laboratory tests, imaging findings, treatment strategies, and prognoses. Additionally, we summarized data from 33 confirmed SMPP-related ICT cases reported in 12 references.

**Results:**

In our cohort, the ICT group exhibited higher incidences of tachypnea, chest pain, inflammation, and elevated D-dimer levels. They also presented more severe radiological findings and had longer hospital stays compared to the non-ICT group. The chordae tendineae-attached was the most common type (61.1%). Pathological examinations revealed ICT sizes ranging from 480 to 31,500 mm³. A favorable prognosis was observed in 94.4% of ICT patients. Clinical features did not significantly differ between various ICT types. In the overall cohort (51 cases), the right ventricle was the predominant location (68.6%). Notably, 66.7% of patients had concurrent extracardiac thrombosis, with pulmonary thrombosis being the most common subtype (41.2%).

**Conclusions:**

The clinical characteristics of SMPP-related ICT are non-specific, often coexisting with severe pulmonary lesions and significantly elevated inflammatory markers. All ICT types were chordae tendineae or wall-attached, rather than mobile. These findings suggest that an inflammation storm induced by SMPP may play a significant role in the pathogenesis of in situ thrombosis within the heart and major blood vessels.

**Supplementary Information:**

The online version contains supplementary material available at 10.1186/s13052-025-01890-3.

## Introduction

*Mycoplasma pneumoniae* (MP) pneumonia (MPP) accounts for 10–40% of community-acquired pneumonia (CAP) cases [1–3], particularly affecting children aged 5 years and older [4]. In certain regions of China, the incidence of MPP can reach up to 70% [5]. Severe MPP (SMPP) presents significant challenges due to its potential to cause serious pulmonary and extrapulmonary complications [6, 7]. Notably, among the extrapulmonary complications of SMPP, the cardiovascular system commonly manifests myocarditis, pericarditis, and arrhythmias. Intracardiac thrombus (ICT) remains one of the rarest types of cardiovascular complications. Additionally, thrombotic diseases are among the most serious complications of MPP, potentially becoming life-threatening if not promptly diagnosed and treated. Thrombotic diseases can affect various organs, including the lungs, brain, spleen, and peripheral blood vessels. ICT stands out as one of the rarest thrombotic complications as well. In this study, we present findings from 18 pediatric patients with SMPP-related ICT and review an additional 33 confirmed cases reported worldwide between 2006 and 2024. To the best of our knowledge, this represents the most extensive and comprehensive investigation into the clinical characteristics of SMPP-related ICT to date.

## Methods

### Subjects

We retrospectively enrolled consecutive patients under 18 years of age who were referred to the Respiratory Department of the Children’s Hospital Affiliated to Zhengzhou University from January 2014 to January 2024, and met the diagnostic criteria for SMPP-related ICT. Contemporaneous SMPP patients without ICT, matched for age and sex, were included as a control group. This study was approved by the Ethics Committee of Children’s Hospital Affiliated to Zhengzhou University (2024-k-046). Informed written consent was obtained from all participants or their parent/legal guardians. MP infection was confirmed by either a fourfold rise in cold agglutinin titer from the acute to the convalescent phase and/or positive polymerase chain reaction (PCR) testing of sputum/bronchoalveolar lavage fluid (BALF). SMPP is defined as MPP that meets the criteria for severe community-acquired pneumonia according to the Chinese Pediatric MPP Diagnosis and Treatment Guidelines (2023 version) [8]. The diagnosis of ICT was established using echocardiography and cardiac computed tomography angiography (CTA), with post-thrombectomy histopathology confirming the diagnosis. We excluded patients with neuromuscular, hematological, or genetic underlying disorders; congenital airway malformations or chronic lung diseases; co-infections with other pathogens; and recent pharmacologic treatments (including aspirin, nonsteroidal anti-inflammatory drugs, and corticosteroids).

### Clinical evaluation

We collected clinical data from all enrolled patients, encompassing demographic information, venous thromboembolism (VTE) and cardiovascular disease (CVD) histories, disease durations, hospitalization durations, clinical symptoms (including fever, cough, tachypnea, dyspnea, chest pain, and hemoptysis), sites and types of ICT, sites of extracardiac concomitant thrombosis (ECT), treatment strategies, and prognoses. Laboratory examinations included inflammatory markers (white blood count [WBC], C-reactive protein [CRP], interleukin [IL]-6, erythrocyte sedimentation rate [ESR], procalcitonin [PCT], lactate dehydrogenase [LDH], and serum ferritin [SF]) and coagulation indicators (prothrombin time [PT], activated partial thromboplastin time [APTT], fibrinogen [FIB], and D-dimer). Imaging modalities such as chest computed tomography (CT) scans, cardiac CTA, echocardiography, bronchoscopy, and abdominal ultrasound were performed to evaluate pulmonary diseases and extrapulmonary complications. Pathogen identification involved sputum/BALF cultures, as well as serological and PCR tests for MP. Post-discharge follow-up occurred monthly. Patients whose ICT resolved spontaneously or was surgically removed were followed for 6 months after discharge. Patients with persistent ICT at discharge were monitored until 6 months after thrombus resolution.

### Review of the literature

We conducted a comprehensive review of all published cases of SMPP complicated by ICT worldwide. Our search spanned databases such as China National Knowledge Infrastructure, Wanfang, PubMed, EMBASE, Cochrane Library, OVID Medicine, and SinoMed, covering the period from January 2006 to January 2024. The search strategy included the following term keys: (“*Mycoplasma pneumoniae* pneumonia”) AND (“intracardiac thrombus”) OR (“thrombosis”). We analyzed various study types, including meta-analyses, case reports, case series, and reviews. In our final analysis, we included original articles that met specific criteria, while excluding duplicate reports. Information about each patient was then compiled, including manifestations, diagnostic information, and treatment attempts. To reduce publication bias and account for nonrandom missing data, only clearly stated manifestations were included as part of the phenotype, with unlisted manifestations assumed to be absent.

### Statistical analysis

Statistical analyses were performed using SPSS software (version 22.0; SPSS, Chicago, IL, USA). Data were expressed as mean ± standard deviation (SD) or median (interquartile range, IQR), depending on the distribution unless otherwise specified. An independent sample t-test was used for continuous variables, while the χ2 test (with Yates correction) was used for categorical variables. If the data could not be transformed to approximate a normal distribution, the Mann-Whitney U test was applied. Binary logistic regression analysis was employed to evaluate significant risk factors for ICT in SMPP. A p value of less than 0.05 (two-tailed) was considered statistically significant.

## Results

### Clinical description of our cohort

We enrolled 18 pediatric patients (14 males and 4 females) who me the inclusion criteria for SMPP-related ICT. A comparison of clinical characteristics, laboratory and imaging examinations, and outcomes between the ICT and non-ICT groups is presented in Table 1. Except for 2 patients (aged 8.0 and 10.0 years) with a patent foramen ovale, none of the patients in either group had a history of VTE or CVD. Fever and cough were the most common symptoms in both groups. There was no statistical difference between the groups with respect to the rate of dyspnea, hemoptysis, and cardiopulmonary failure. However, the ICT group had a significantly higher incidence of tachypnea and chest pain compared to the non-ICT group. In the ICT group, 6 children (33.3%) exhibited no thrombosis-related symptoms, presenting only with pneumonia symptoms of fever and cough. The mean time of ICT detection was 12.9 ± 3.7 days, ranging from 7 to 20 days.

**Table 1:** Comparison of clinical features between SMPP-related ICT patients and non-ICT patients in this study, as well as between ICT patients in this study and all reported ICT cases in the literature

Variables	Total ICT reported (Group 1, *n* = 51)	ICT in this study (Group 2, *n* = 18)	Non-ICT in this study (Group 3, *n* = 50)	*p* value (Group 1 vs. 2)	*p* value (Group 2 vs. 3)
Age, year					
Median (IQR)	7.0 (6.0, 9.0)	8.0 (7.0, 9.6)	7.5 (6.0, 10.0)	0.781	0.748
Range	3.9–52.0	3.9–13.0	4.0–12.0	NA	NA
Sex, male/female	30/13	14/4	39/11	0.755	1.000
VTE history, n (%)	0 (0)	0 (0)	0 (0)	NA	NA
CVDs history, n (%)	2 (3.9)	2 (11.1)	0 (0)	0.277	NA
Disease duration at ICT Dx, day					
Mean ± SD	NA	12.9 ± 3.7	NA	NA	NA
Range		7.0–20.0			
Hospitalization duration, day					
Mean ± SD	NA	29.1 ± 8.9	12.58 ± 3.1	NA	**<0.001**
Range		13.0–49.0	8.0–12.0		NA
**Clinical manifestations**,** n (%)**					
Fever	51 (100.0)	18 (100.0)	50 (100.0)	NA	NA
Tmax, ℃	NA	39.7 ± 0.7	39.7 ± 0.6	NA	0.929
Cough	51 (100.0)	18 (100.0)	50 (100.0)	NA	NA
Tachypnea	17 (33.3)	10 (55.6)	7 (14.0)	**0.039**	**<0.001**
Dyspnea	6 (11.8)	3 (16.7)	13 (26.0)	0.687	0.529
Hemoptysis	1 (2.0)	1 (5.6)	0 (0)	0.457	0.265
Chest pain	17 (33.3)	9 (50.0)	5 (10.0)	**<0.001**	**<0.001**
Cardiopulmonary failure	1 (2.0)	1 (5.6)	0 (0)	0.457	0.265
**Types of ICT**,** n (%)**					
Mobile type	NA	0 (0)	NA	NA	NA
Chordae tendineae-attached type		11 (61.1)			
Wall-attached type		6 (33.3)			
Obstructive type		1 (5.6)			
**Sites of ICT**,** n (%)**					
Left atrium	3 (5.9)	0 (0)	NA	0.562	NA
Right atrium	8 (15.7)	4 (22.2)		0.497	
Left ventricle	4 (7.8)	1 (5.6)		1.000	
Right ventricle	35 (68.6)	13 (72.2)		1.000	
Aortic arch	1 (2.0)	0 (0)		1.000	
**Sites of ECT**,** n (%)**					
None	17 (33.3)	8 (44.4)	50 (100.0)	0.409	**<0.001**
Pulmonary thrombosis	21 (41.2)	9 (50.0)	0 (0)	0.586	NA
Splenic thrombosis	2 (3.9)	1 (5.6)	0 (0)	0.182	NA
Renal thrombosis	2 (3.9)	1 (5.6)	0 (0)	1.000	NA
Cerebral thrombosis	2 (3.9)	0 (0)	0 (0)	1.000	NA
Others	2 (3.9)	0 (0)	0 (0)	1.000	NA
**Laboratory parameters**					
WBC, ×10^9^/L	NA	11.5 ± 2.8	8.5 ± 2.6	NA	**<0.001**
Neutrophil percentage, %		82.1 ± 6.6	67.8 ± 9.5		**<0.001**
PLT, ×10^9^/L		315.2 ± 112.9	292.5 ± 108.5		0.461
CRP, mg/L		46.7 (14.3, 59.8)	18.9 (10.1, 43.5)		**<0.001**
IL-6, pg/mL		32.7 (16.3, 64.5)	27.0 (11.5, 49.9)		0.288
ESR, mm/h		34.0 (17.0, 53)	45.0 (33.3, 69.0)		0.057
PCT, ng/mL		0.3 (0.1, 0.4)	0.2 (0.1, 0.4)		0.961
LDH, U/L		729.3 (541.0, 865.0)	342.5 (290.8, 468.8)		**<0.001**
SF, ng/mL		347.5 (183.6, 590.0)	134.2 (90.2, 225.3)		**<0.001**
PT, s		12.8 ± 0.9	14.3 ± 2.5		**<0.001**
APTT, s		25.0 ± 4.4	29.3 ± 18.1		0.333
Fibrinogen, g/L		3.9 ± 1.8	3.8 ± 0.9		0.925
D-Dimer, ug/mL		5.3 (3.6, 7.4)	0.8 (0.5, 1.7)		**<0.001**
≥ 5 µg/mL, n (%)		10 (55.6)	2 (4.0)		**<0.001**
<5 µg/mL, n (%)		8 (44.4)	48 (96.0)		**<0.001**
Positive Anti-aCL antibodies, n (%)		0 (0)	0 (0)		NA
**Chest CT imaging**,** n (%)**					
High-density consolidation (≥ 2/3 single lobe)	NA	18 (100.0)	25 (50.0)	NA	**<0.001**
Necrotizing pneumonia		9 (50.0)	13 (26.0)		0.081
Pleural effusion		8 (44.4)	21 (42.0)		1.000
**Therapeutic agents**,** n (%)**					
Anticoagulation therapy	30 (58.8)	18 (100.0)	NA	**<0.001**	NA
Low molecular weight heparin	NA	17 (94.4)		NA	
Warfarin	NA	0 (0)		NA	
Rivaroxaban	NA	1 (5.6)		NA	
Thrombolysis therapy	5 (9.8)	0 (0)		0.316	
Surgical thrombectomy	16 (31.4)	13 (72.2)		**0.005**	
**Outcomes**,** n (%)**					
Recovered/Improved	NA	17 (94.4)	42(84.0)	NA	0.427
Died		0 (0)	0 (0)		NA
Lost to follow-up		1 (5.6)	8 (16.0)		0.427

Bold values represent *p* < 0.05

**Abbreviations**:

aCL: Anti-cardiolipin; APTT: Activated partial thromboplastin time; CRP: C-reactive protein; CVD: Cardiovascular disease; ESR: Erythrocyte sedimentation rate; ICT: Intracardiac thrombus; IL: Interleukin; LDH: Lactate dehydrogenase; PCT: Procalcitonin; PLT: Platelets; PT: Prothrombin time; SF: Serum ferritin; VTE: Venous thromboembolism; WBC: White blood count

The ICT group showed elevated levels of WBC, neutrophil percentage, CRP, LDH, SF, and D-dimer, and a decreased levels of PT compared with the non-ICT group. However, no significant intergroup differences were observed in platelet count, IL-6, ESR, PCT, APTT, and FIB. In the ICT group, D-dimer levels were nonspecific: 44.4% were < 5 µg/mL, and 55.6% were ≥ 5 µg/mL, with a maximum value of 53.3 µg/mL. Nine patients underwent screening for anti-cardiolipin (aCL) antibodies, and none tested positive. Additionally, the ICT group had more severe radiological findings, evidenced by a significantly higher rate of consolidation involving ≥ 2/3 of a lobe, and experienced longer hospital stays compared to the non-ICT group.

**Fig. 1:**
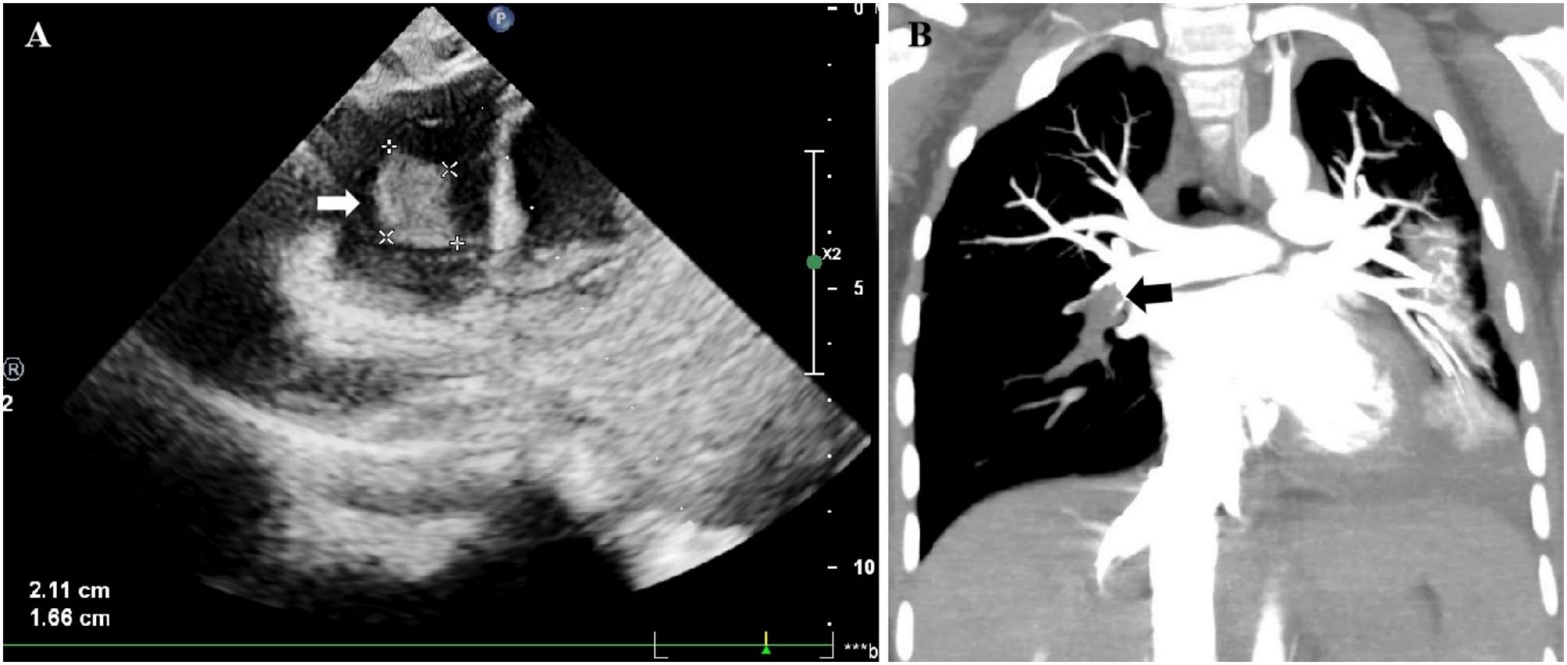
**A**, Echocardiography revealed a wall-attached mass (indicated by the white arrow) in the right ventricle. **B**, Enhanced Chest CT demonstrated a low-density filling defect in the lower right pulmonary artery (indicated by the black arrow), suggestive of pulmonary thrombosis

Among the 18 children with ICT, the right ventricle was the most frequent site of ICT (13 cases, 72.2%) (Fig. 1A), followed by the right atrium (4 cases, 22.2%). Among the different types of ICT, the chordae tendineae-attached type was the most common (11 cases, 61.1%), followed by the wall-attached type (6 cases, 33.3%) and the obstructive type (1 case, 5.6%). Interestingly, the mobile type was not observed in any cases. A comparison of the clinical characteristics, sites of ICT, treatment strategies, and prognostic outcomes of patients with different types of ICT is shown in Table 2. The results showed no significant differencees between the two groups. Regarding ECT, 8 (44.4%) did not exhibit any ECT, while 9 (52.9%) simultaneously had ECT, with pulmonary thrombosis being the most common subtype (50.0%) (Fig. 1B).

**Table 2:** Comparison of clinical features of patients with different types of ICT

Variables	Chordae tendineae-attached ICT (*n* = 11)	Wall-attached ICT (*n* = 6)	*p* value
Age, year	8.0 (7.0, 9.2)	6.4 (4.8, 7.6)	0.106
Sex, male/female	8/3	6/0	0.515
VTE history, n (%)	0 (0)	0 (0)	NA
CVDs history, n (%)	1 (9.1)	0 (0)	1.000
Disease duration at ICT Dx, day	13.7 ± 3.1	12.0 ± 4.4	0.312
Hospitalization duration, day	29.2 ± 10.3	29.8 ± 2.5	0.481
**Clinical manifestations**,** n (%)**			
Fever	11 (100.0)	6 (100.0)	NA
Cough	11 (100.0)	6 (100.0)	NA
Tachypnea	6 (54.5)	3 (50.0)	1.000
Dyspnea	1 (9.1)	1 (16.7)	1.000
Hemoptysis	1 (9.1)	0 (0)	1.000
Chest pain	7 (63.6)	2 (33.3)	0.335
Cardiopulmonary failure	0 (0)	0 (0)	NA
**Sites of ICT**,** n (%)**			
Left atrium	0 (0)	0 (0)	NA
Right atrium	1 (9.1)	3 (50.0)	0.099
Left ventricle	1 (9.1)	0 (0)	1.000
Right ventricle	9 (81.8)	3 (50.0)	0.280
Aortic arch	0 (0)	0 (0)	NA
**Therapeutic agents**,** n (%)**			
Anticoagulation therapy	11 (100.0)	6 (100.0)	NA
Thrombolysis therapy	0 (0)	0 (0)	NA
Surgical thrombectomy	6 (54.5)	5 (83.3)	0.333
**Outcomes**,** n (%)**			
Recovered	10 (90.9)	6 (100.0)	1.000
Died	0 (0)	0 (0)	NA
Lost to follow-up	1 (9.1)	0 (0)	1.000

Abbreviations:

CVD: Cardiovascular disease; ICT: Intracardiac thrombus; VTE: Venous thromboembolism

**Fig. 2:**
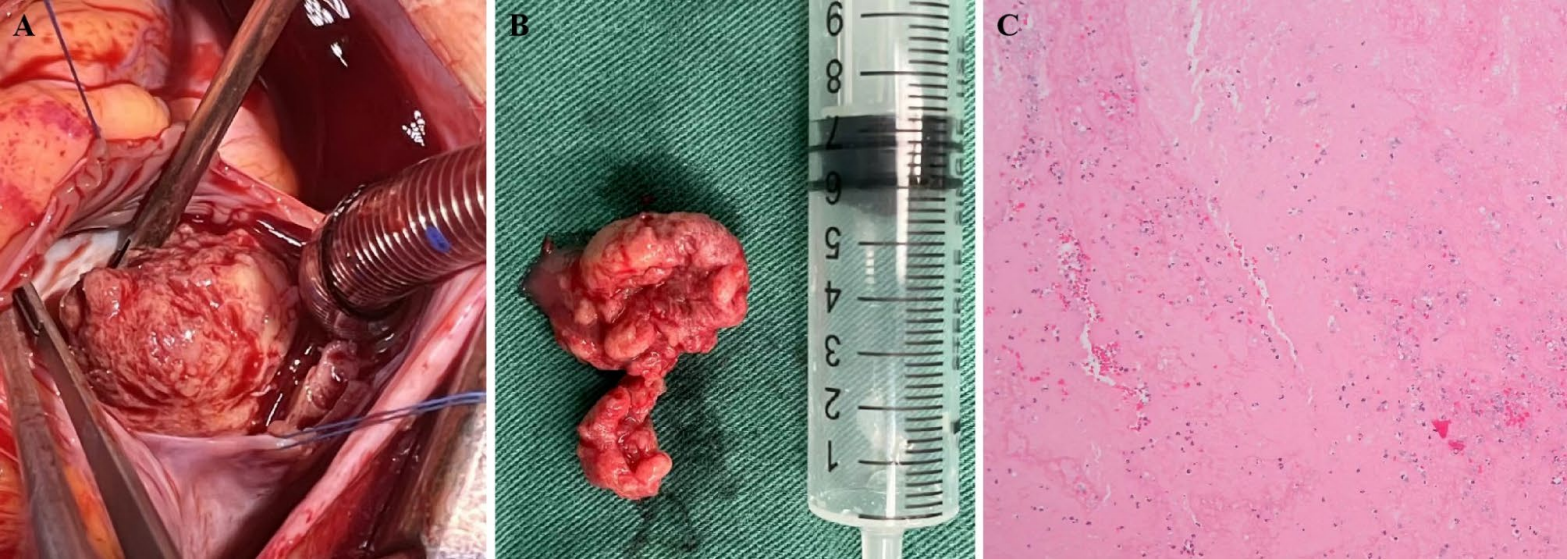
Intraoperative and pathological features of the mass. **A**,** B**, During intraoperative and gross examination, a thrombus measuring 17.4 × 14.2 mm was found attached to the right ventricular wall. **C**, Hematoxylin-eosin staining (at 100x magnification) revealed abundant red blood cells, dispersed neutrophils, lymphocytes, and tissue cells within the fibrinous exudate

All 18 patients with ICT received anti-inflammatory glucosteroids and anticoagulation therapy. Of these, 13 (72.2%) underwent surgical thrombectomy. Notably, Case 51 experienced severe cardiopulmonary failure. Despite pure oxygen mechanical ventilation, the oxygenation index (PaO_2_/FiO_2_) remained below 60 mmHg for over 3 hours. This patient underwent extracorporeal membrane oxygenation therapy and emergency surgical thrombectomy. Gross pathologic examinations revealed blood clots ranging from 480 to 31,500 mm^3^ in size. Hematoxylin-eosin staining (at 100x magnification) revealed abundant red blood cells, dispersed neutrophils, lymphocytes, and tissue cells within the fibrinous exudate (Fig. 2). Among the 5 patients who did not undergo surgery, two experienced spontaneous thrombus resolution during hospitalization, two had their thrombus resolved during follow-up at 3 months and 4 months, respectively. One patient had unresolved thrombosis at discharge but was subsequently lost to follow-up. Fortunately, in all follow-up patients, there were no occurrences of death or recurrent thrombosis.

### Risk factors for ICT development in SMPP

We conducted a binary logistic regression analysis with 9 independent variables (chest pain, tachypnea, WBC, CRP, LDH, SF, PT, D-dimer, and consolidation involving ≥ 2/3 of a single lobe) that showed significant differences in the univariate analysis. However, the results were negative, indicating that none of the potential risk factors we examined significantly predicted the development of ICT in SMPP.

### Review of clinical manifestations (present cohort and literature data)

Based on a review of the literature, an additional 33 patients described in 12 references were included (Mainland China [28 cases], India [1 Case], Japan [1 Case], France [1 Case], Australia [1 Case], and the USA [1 case]). A total of 51 patients with SMPP-related ICT were included in the final analyses. Demographic data and clinical manifestations of this population are shown in Table 3.

**Table 3:** Cases of *Mycoplasma pneumoniae* pneumonia complicated with intracardiac thrombus reported in the literature

Case	References	Age, y	Sex	Main symptoms	Size, mm	Sites of ICT	Sites of ECT	Therapy
1	2006 [9]	4.0	M	1/2	NA	Left atrium	None	A
2	2010 [10]	8.0	M	1/2	NA	Right ventricle	None	A + S
3	2013 [11]	41.0	M	1/2	16.0 × 9.0	Aortic arch	Splenic, renal and peroneal artery	A
4	2015 [12]	52.0	F	1/2/3/4/6	30.0 × 34.0	Left ventricle	None	A + S
5	2018 [13]	6.0	M	1/2	17.0 × 9.3	Right ventricle	None	A + S
6	2018 [13]	6.0	M	1/2	15.0 × 10.0	Right atrium	None	A
7	2021 [14]	37.0	M	1/2/3/4/6	NA	Left ventricle	Left middle cerebral artery	A
8	2021 [15]	5.0	M	1/2	NA	Left atrium	Right middle cerebral artery	A
9	2021 [16]	5.0	F	1/2/6	1.3 × 0.9	Right ventricle	Right pulmonary artery (Middle lobe branch)	A
10	2021 [16]	9.0	M	1/2/3	1.6 × 1.6	Right ventricle	None	A
11	2021 [16]	5.0	F	1/2/3	0.8 × 0.5	Right ventricle	Right pulmonary artery	A
12	2021 [16]	3.0	M	1/2	1.1 × 0.9	Right ventricle	Bilateral pulmonary artery (inferior lobe branch)	A
13	2021 [16]	8.0	M	1/2/3	2.8 × 1.8	Right ventricle	Pulmonary artery	A
14	2021 [16]	6.0	F	1/2/3	0.7 × 0.5	Right ventricle	None	A
15	2021 [16]	7.0	M	1/2	1.3 × 0.8	Right ventricle	Right pulmonary artery (inferior lobe branch)	A
16	2021 [16]	13.0	M	1/2/3	1.4 × 1.1	Right ventricle	Left pulmonary artery (superior lobe branch)/Right pulmonary artery (middle lobe branch)	A
17	2021 [16]	6.0	M	1/2	1.5 × 0.9	Right ventricle	Right pulmonary artery (inferior lobe branch)	A
18	2021 [16]	7.0	M	1/2	0.6 × 0.2	Right ventricle	None	A
19	2022 [17]	8.0	F	1/2/4/6	4.0 × 3.0/13.0 × 5.0/9.0 × 5.0	Right ventricle	Left pulmonary artery (inferior lobe branch)	T
20	2022 [18]	8.0	F	1/2/3	13.0 × 11.0	Right ventricle	Left pulmonary artery (inferior lobe branch)	T
21	2022 [18]	7.0	M	1/2/6	15.0 × 11.0	Right atrium	Left pulmonary artery (inferior lobe branch)	T
22–26	2023 [19]	NA	NA	NA	NA	Right ventricle	NA	A
27	2023 [19]	NA	NA	NA	NA	Left ventricle	NA	A
28	2023 [19]	NA	NA	NA	NA	Left atrium	NA	A
29	2023^[19]^	NA	NA	NA	NA	Right atrium	NA	A
30	2023^[20]^	12.0	F	1/2/6	NA	Right ventricle	Right deep femoral artery/Right posterior tibial artery/Right dorsal pedal artery	A
31	2023^[20]^	8.0	F	1/2/6	NA	Right ventricle	Left lower pulmonary artery	T
32	2023^[20]^	9.0	F	1/2/6	NA	Right ventricle	None	A
33	2023^[20]^	7.0	M	1/2/6	NA	Right atrium	Right pulmonary artery (inferior lobe branch)	T
34	This study	8.0	M	1/2/3/6	13.6 × 11.1	Right ventricle	Right pulmonary artery (inferior lobe branch)	A
35	This study	12.0	M	1/2	12.6 × 8.7	Right ventricle	Bilateral pulmonary artery (inferior lobe branch)	A
36	This study	7.0	M	1/2	40.0 × 15.0	Right ventricle	Left pulmonary artery (inferior lobe branch)	A + S
37	This study	4.5	M	1/2	10.5 × 9.3	Right ventricle	Right pulmonary artery (inferior lobe branch)	A
38	This study	12.0	M	1/2/3/6	32.4 × 17.9	Right ventricle	None	A + S
39	This study	5.7	M	1/2/3/6	14.6 × 7.8	Right ventricle	None	A + S
40	This study	4.3	F	1/2/3	16.5 × 11.2	Right ventricle	Left pulmonary artery (superior lobe branch)/Right pulmonary artery (inferior lobe branch)	A + S
41	This study	3.9	M	1/2/3/4	17.4 × 14.2	Right ventricle	None	A + S
42	This study	8.7	M	1/2/6	9.6 × 9.5	Right ventricle	Left pulmonary artery (inferior lobe branch)	A + S
43	This study	8.0	M	1/2/6	7.6 × 5.2	Right ventricle	None	A
44	This study	7.0	M	1/2	16.1 × 11.0	Right ventricle	None	A
45	This study	7.6	F	1/2/6	10.1 × 6.4	Right ventricle	Right pulmonary artery (inferior lobe branch)	A + S
46	This study	13.0	F	1/2/3/6	8.0 × 5.0	Left ventricle	Splenic and renal artery	A + S
47	This study	9.7	M	1/2	15.7 × 14.6	Right atrium	None	A + S
48	This study	9.2	M	1/2/3/4/5/6	19.4 × 10.9	Right atrium	Right pulmonary artery (inferior lobe branch)	A + S
49	This study	8.0	M	1/2/3	9.7 × 8.7	Right atrium	None	A + S
50	This study	7.0	M	1/2/3/6	16.4 × 13.9	Right atrium	None	A + S
51	This study	10.0	F	1/2/3/4	2.5 × 2.0	Right ventricle	Right pulmonary artery (inferior lobe branch)	A + S

Abbreviations

Sex: F: Female; M: Male

Main symptoms: 1: Fever; 2: Cough; 3: Tachypnea; 4: Dyspnea; 5: Hemoptysis; 6: Chest pain

Site: ICT: Intracardiac thrombus; ECT: Extracardiac thrombus; PT: Pulmonary thrombosis

Therapeutic plans: T: Thrombolytic therapy; A: Anticoagulant therapy; S: Surgical thrombectomy

The ages of these patients ranged from 3.9 to 52.0 years, with a media age of 8.0 years. The male-to-female ratio was 3.5:1. The most frequently reported symptoms were fever and cough (100% each). Additionally, tachypnea and chest pain were observed in 55.6% and 50% of cases, respectively. Among the 51 patients, the right ventricle was the most frequent site of ICT (68.6%), followed by the right atrium (15.7%). The left ventricle, left atrium, and aortic arch were less commonly affected, representing 7.8%, 5.9%, and 2.0% of cases, respectively. Regarding ECT, 66.7% of patients simultaneously had ECT, with pulmonary thrombosis being the most common type (41.2%). In terms of treatment options, 58.8% of patients underwent anticoagulation therapy, 31.4% underwent surgical thrombectomy, and 9.8% received thrombolysis therapy. A comparison of clinical features between ICT patients in this study and all reported ICT cases in the literature is shown in Table 1.

## Discussion

The incidence rate of SMPP-related ICT remains unreported to date. Currently, only 51 cases (including our cohort of 18 cases) were documented [9–20] (Table 3). Our research confirms that the clinical features of SMPP-related ICT lack specificity, with symptom severity varying depending on the size, location, and activity of the thrombus. While mild cases may be asymptomatic or present only with pneumonia-like symptoms, severe cases can lead to cardiopulmonary failure (Case 51 in Table 3), or even sudden death. Thrombus detachment can occur via either the pulmonary or systemic circulation, resulting in embolization to critical organs such as the lungs, brain, kidneys, and spleen. Consequently, the mortality associated with SMPP-related ICT is alarmingly high if not promptly treated.

Our study confirms that the right cardiac chambers are the most common site for ICT, accounting for 84.3% of all cases. According to data from the European Society of Cardiology, the presence of a mobile thrombus in the right cardiac chambers is associated with an unfavorable outcome, with mortality rates ranging from 80 to 100% [21]. However, our study yielded different results. All patients in our cohort had favorable prognoses, and there were no fatalities. The explanation for this discrepancy may lie in the different types of ICT. In our study, SMPP-related ICT cases predominantly exhibited either the chordae tendineae-attached type (61.1%) or the wall-attached type (33.3%), with no instances of the mobile type (as shown in Table 1). This study provides the first evidence that the mechanism underlying ICT related to MPP is not systemic emboli detachment, which typically causes classic ICT. Instead, it appears to involve in situ thrombosis within the heart and major blood vessels.

The pathophysiological mechanisms of SMPP-induced cardiovascular complications remain unclear. These mechanisms can be classified into three categories: direct type (inflammatory cytokines induced by MP lipoproteins causing endocardial injury), indirect type (immune modulation through cross-reaction), and vascular occlusion type (vasculitis and/or thrombosis) [22]. Reports suggest that SMPP-induced vasculitis/thrombosis involves autoimmune reactions and molecular mimicry, promoting the production of antibodies against phospholipids, glycerides, and proteins, resulting in associated vasculitic/thrombotic disorders regardless of a systemic hypercoagulable state [22, 23]. The “pro-thrombotic” effect of anti-phospholipid antibodies is linked to several mechanisms, including cellular activation (direct stimulation of endothelial cell procoagulant activity and enhancement of platelet activation and aggregation), inhibition of endogenous anticoagulants (such as protein C, protein S, anti-thrombin, and annexin A5) [24, 25], impairment of fibrinolysis, and complement activation. Witmer. et al. [26] and Ortel. et al. [27] reported that over 50% of patients with MPP combined with thrombosis tested positive for aCL-IgM, which gradually disappeared during the recovery phase. However, in this study, nine patients were screened for aCL antibodies, and none tested positive.

It’s worth noting that coagulation tests do not definitively indicate a hypercoagulable state. In our study, PT, APTT and fibrinogen levels were all within the normal range. D-dimer levels showed nonspecific results: 44.4% were < 5 µg/mL, and 55.6% were ≥ 5 µg/mL, with a maximum value of 53.25 µg/mL (Case 51 in Table 3). Case 51 experienced extensive pulmonary thrombosis, affecting the right middle and lower lobes, due to partial detachment of a right ventricular thrombus. Additionally, Case 46 had a D-dimer level of 19.6 µg/mL and presented with multi-organ thrombosis, including involvement of the heart, spleen, and kidney. Our findings suggest that while higher D-dimer levels are strongly associated with SMPP-related thrombotic disorders, normal D-dimer levels do not definitively exclude the possibility of thrombotic events.

The diagnosis of SMPP-related ICT primarily relies on echocardiography [28]. This imaging technique reveals the thrombus’s morphology, location, size, activity, as well as cardiac function and blood flow. Echocardiography demonstrates high sensitivity and specificity for diagnosing SMPP-related ICT, making it the preferred non-invasive diagnostic method. However, it’s essential to differentiate SMPP-related ICT from other conditions, such as infective endocarditis or cardiac tumors with associated infections, which can also present with fever, elevated inflammatory markers, and the formation of intracardiac masses or tumors. Infective endocarditis frequently affects the left heart and presents as high fever, cardiac murmur, and peripheral thrombosis [29]. The prevalent pathogen is gram-positive cocci. Blood or vegetation culture and pathological examination can assist in differentiation. Cardiac tumors in children are extremely rare. The use of contrast echocardiography and cardiac magnetic resonance imaging in the assessment of cardiac masses has been shown to be helpful in distinguishing tumors from thrombi [30].

The treatment of ICT lacks standardized guidelines and typically involves anticoagulation, thrombolytic therapy, and surgical thrombectomy. Each approach has its limitations and advantages. Anticoagulation serves as the foundational treatment. Commonly used anticoagulants include low-molecular-weight heparin, vitamin K antagonists, and novel oral anticoagulants such as rivaroxaban and apixaban, which are now being applied in pediatric cases [31, 32]. Thrombolytic therapy is most effective when administered early during thrombus formation. However, the specific time window requires further research. The primary complication associated with thrombolytic therapy is the risk of bleeding, necessitating a thorough assessment of bleeding tendencies before initiating treatment. Recombinant tissue-type plasminogen activator (rt-PA) is the preferred thrombolytic agent. Dong. et al. [18] have reported successful outcomes with thrombolytic therapy in cases of MPP complicated by pulmonary and intracardiac thrombi. However, further research is needed to clarify the indications, administration routes, dosages, and duration of thrombolytic therapy. Currently, surgical thrombectomy remains a topic of ongoing debate regarding timing and necessity. In our cohort, 72.2% underwent combined surgical and anticoagulation therapy, while 27.8% received conservative anticoagulation therapy only. Except for one lost-to-follow-up case, all other patients achieved favorable treatment outcomes, with no recurrence of thrombotic diseases or cardiac-related sequelae. Our patients’ prognosis aligns with reports by Li. et al. [13] and Fu. et al. [20]. Therefore, for pediatric patients with SMPP-related ICT, the indications for thrombectomy remain unclear. Decisions regarding thrombectomy should take into account factors such as thrombus size, location, activity, the possibility of conservative treatment, cardiopulmonary reserve, and comorbidities.

## Conclusions

ICT is one of the rarest cardiac complications associated with SMPP and also represents the rarest type of thrombotic disorder in SMPP. The clinical features of SMPP-related ICT lack specificity; they often coincide with severe pulmonary lesions and significantly elevated inflammatory markers. These findings suggest that an inflammation storm induced by SMPP may play a significant role in the pathogenesis of thrombosis formation. Additionally, the right ventricle emerged as the predominant location, and the chordae tendineae-attached was the most common ICT type. These findings provide the first evidence that the mechanism underlying ICT related to MPP is not systemic emboli detachment, which typically causes classic ICT. Instead, it appears to involve in situ thrombosis within the heart and major blood vessels. Anticoagulation therapy is the preferred treatment, while the decision for surgical thrombectomy should consider multiple factors. Early diagnosis and intervention lead to favorable long-term outcomes for ICT patients.

## Electronic supplementary material

Below is the link to the electronic supplementary material.

Supplementary Material 1

## Data Availability

Data sharing not applicable to this article as no datasets were generated or analyzed during the current study.

## Abbreviations

aCL: Anti-cardiolipin
APTT: Activated partial thromboplastin time
CRP: C-reactive protein
CVD: Cardiovascular disease
ICT: Intracardiac thrombus
LDH: Lactate dehydrogenase
MP: Mycoplasma pneumoniae
MPP: Mycoplasma pneumoniae pneumonia
PT: Prothrombin time
SF: Serum ferritin
SMPP: Severe Mycoplasma pneumoniae pneumonia
VTE: Venous thromboembolism
WBC: White blood count
