# Supplementary material for: Clinical features of intracardiac thrombotic complication in patients with severe Mycoplasma pneumoniae pneumonia
Source: Ital J Pediatr. 2025 Feb 11;51:42. doi: 10.1186/s13052-025-01890-3 (PMC11817218; doi:10.1186/s13052-025-01890-3)
Supplement: Supplementary file 1 — Supplementary Material 1 [file 13052_2025_1890_MOESM1_ESM.pdf]

SPRINGER NATURE

Change of authorship request form - Journals (pre-acceptance)

Important information. Please read.

- This form should be used by authors to request any change in authorship (adding/deleting authors) including changes in corresponding authors. This form should not be used for name changes. Please fully complete all sections. Use black ink and block capitals and provide each author's full name with the given name first followed by the family name.

- By signing this declaration, all authors guarantee that the order of the authors are in accordance with their scientific contribution, if applicable as different conventions apply per discipline, and that only authors have been added who made a meaningful contribution to the work.

- Please note, in author collaborations where there is formal agreement for representing the collaboration, it is sufficient for the representative or legal guarantor (usually the corresponding author) to complete and sign the Authorship Change Form on behalf of all authors, **next to the added/removed author(s)**. **(Complete Section 3, followed by Section 6.)**

  In author collaborations where there is no formal agreement for representing the collaboration and **there are more than 10 authors**, one may sign for all, provided the signer appends correspondence that attests that each of the authors have agreed to the change **and the added/removed authors sign the form**. **(Complete Section 3, followed by Section 6.)**

- Please note, we cannot investigate or mediate any authorship disputes. If you are unable to obtain agreement from all authors (including those who you wish to be removed) you must refer the matter to your institution(s) for investigation. Please inform us if you need to do this.

- If you are notable to return a fully completed form within **30 days** of the date that it was sent to the author requesting the change, we may have to withdraw your manuscript. We cannot publish manuscripts where authorship has not been agreed by all authors (including those who have been removed).

- Incomplete forms will be rejected.

- Please return/upload this form, fully completed, to the Journals Editorial Office. The Journal and/or Publisher will consider the information you have provided to decide whether to approve the proposed change in authorship. We may decide to contact your institution for more information or undertake a further investigation, if appropriate, before making a final decision.

SPRINGER NATURE Springer Nature is one of the world's leading global research, educational and professional publishers, created in May 2015 through the combination of Nature Publishing Group, Palgrave Macmillan, Macmillan Education and Springer Science+Business Media.

Change of authorship request form - Journals (pre-acceptance)

SPRINGER NATURE

Section 1: Please provide the current title of manuscript

Manuscript ID no.: ITJP-D-24-00741R1

Title: Clinical features of intracardiac thrombotic complication in patients with severe Mycoplasma pneumoniae pneumonia

Section 2: Please provide the previous authorship, in the order shown on the manuscript before the changes were introduced. Please indicate the corresponding author by adding (CA) behind the name.

	First name(s)	Family name	ORCID or SCOPUS id, if available
1st author	Xiao	Li	
2nd author	Bo	Zhai	
3rd author	Lei	Zhang	
4th author	Jing	Wang	
5th author	Chunna	Xu	
6th author	Lili	Dong	
7th author	Yanqiong	Wang	
8th author	Yanyan	Su	
9th author	Zhen	Dong	
10th author	Yu	Tang	
11th author	Yuelin	Shen (CA)	0009-0007-3206-610X
12th author			

Please use an additional sheet if there are more than 10 authors.

SPRINGER NATURE Springer Nature is one of the world's leading global research, educational and professional publishers, created in May 2015 through the combination of Nature Publishing Group, Palgrave Macmillan, Macmillan Education and Springer Science+Business Media.

Change of authorship request form - Journals (pre-acceptance)

Section 3: Please provide a justification for change. Please use this section to explain your reasons for changing the authorship of your manuscript, e.g. what necessitated the change in authorship? Please refer to the (journal) policy pages for more information about authorship. Please explain why omitted authors were not originally included and/or why authors were removed on the submitted manuscript.

Haiming Yang participated in conceptualizing and designing the study, followed up with the majority of the patients, revised the manuscript, and acquired funding. Therefore, he has been added as a co-corresponding author.

Section 4: Proposed new authorship. Please provide your new authorship list in the order you would like it to appear on the manuscript. Please indicate the corresponding author by adding (CA) behind the name. If the Corresponding Author has changed, please indicate the reason under section 3.

	First name(s)	Family name (this name will appear in full on the final publication and will be searchable in various abstract and indexing databases)	Affiliated institute	E-mail address
1st author	Xiao	Li	Respiratory Department, Children's Hospital Affiliated to Zhengzhou University, Henan Children's hospital, Zhengzhou Children's Hospital	lxluky@163.com
2nd author	Bo	Zhai	Cardiac Surgery Department,Children's Hospital Affiliated to Zhengzhou University, Henan Children's hospital, Zhengzhou Children's Hospital	Zhaibo3000@163.com
3rd author	Yu	Tang	Respiratory Department, Children's Hospital Affiliated to Zhengzhou University, Henan Children's hospital, Zhengzhou Children's Hospital	tangyu010312@sina.com
4th author	Lei	Zhang	Respiratory Department, Children's Hospital Affiliated to Zhengzhou University, Henan Children's hospital, Zhengzhou Children's Hospital	289089692@qq.com
5th author	Jing	Wang	Respiratory Department, Children's Hospital Affiliated to Zhengzhou University, Henan Children's hospital, Zhengzhou Children's Hospital	1403212239@qq.com
6th author	Chunna	Xu	Respiratory Department, Children's Hospital	454365930@qq.com

---
SPRINGER NATURE Springer Nature is one of the world's leading global research, educational and professional publishers, created in May 2015 through the combination of Nature Publishing Group, Palgrave Macmillan, Macmillan Education and Springer Science+Business Media.

			Affiliated to Zhengzhou University, Henan Children's hospital, Zhengzhou Children's Hospital	
7th author	Lili	Dong	Respiratory Department, Children's Hospital Affiliated to Zhengzhou University, Henan Children's hospital, Zhengzhou Children's Hospital	luckydlily@163.com
8th author	Yanqiong	Wang	Respiratory Department, Children's Hospital Affiliated to Zhengzhou University, Henan Children's hospital, Zhengzhou Children's Hospital	woshiwangyanqiong@126.com
9th author	Yanyan	Su	Respiratory Department, Children's Hospital Affiliated to Zhengzhou University, Henan Children's hospital, Zhengzhou Children's Hospital	suyanyan7902@126.com
10th author	Zhen	Dong	Respiratory Department, Children's Hospital Affiliated to Zhengzhou University, Henan Children's hospital, Zhengzhou Children's Hospital	doctordong@163.com
11th author	Haiming	Yang **(CA)**	Respiratory Department II, National Clinical Research Center for Respiratory Diseases, Beijing Children's Hospital, Capital Medical University, National Center for Children's Health	yanghaiming830@163.com
12th author	Yuelin	Shen **(CA)**	Respiratory Department II, National Clinical Research Center for Respiratory Diseases, Beijing Children's Hospital, Capital Medical University, National Center for Children's Health	shenyuelin@hotmail.com

Please use an additional sheet if there are more than 10 authors.

SPRINGER NATURE Springer Nature is one of the world's leading global research, educational and professional publishers, created in May 2015 through the combination of Nature Publishing Group, Palgrave Macmillan, Macmillan Education and Springer Science+Business Media.

Change of authorship request form - Journals (pre-acceptance)

Section 5: Author contribution, Acknowledgement and Disclosures. Please use this section to provide a new disclosure statement and, if appropriate, acknowledge any contributors who have been removed as authors and ensure you state what contribution any new authors made (if applicable per the journal or book (series) policy). Please ensure these are updated in your manuscript - <u>after approval of the change(s)</u> - as our production department will not transfer the information in this form to your manuscript.

New acknowledgements:

The author would like to thank all the patients and their families who took part in this study and all the physicians for their assistance in completing this work.

New Disclosures (financial and non-financial interests, funding):

The author have no conflicts of interest to disclose.

New Author Contributions statement (if applicable per the journal policy):

Haiming Yang: Conceptualizing & designing, Writing-review & editing, Funding acquisition.

State 'Not applicable' if there are no new authors.

SPRINGER NATURE Springer Nature is one of the world's leading global research, educational and professional publishers, created in May 2015 through the combination of Nature Publishing Group, Palgrave Macmillan, Macmillan Education and Springer Science+Business Media.

SPRINGER NATURE

Change of authorship request form - Journals (pre-acceptance)

Section 6: Declaration of agreement. *All* authors, unchanged, new and removed *must* sign this declaration.
(NB: Please print the form, (docu)-sign and return/upload a scanned copy. Please note that signatures that have been inserted as an imagefile are acceptable as long as it is handwritten. Typed names in the signature box are unacceptable.) *** Please delete as appropriate. Delete all of the bold if you were on the original authorship list and are remaining as an author.**

	First name	Family name		Signature	Date
1st author	Xiao	Li	I agree to the proposed new authorship shown in section 4 /and the proposed change in corresponding author	Xiao Li	5-Jan-2025
2nd author	Bo	Zhai	I agree to the proposed new authorship shown in section 4 /and the proposed change in corresponding author	Bo Zhai	5-Jan-2025
3rd author	Yu	Tang	I agree to the proposed new authorship shown in section 4 /and the proposed change in corresponding author	Yu Tang	5-Jan-2025
4th authors	Lei	Zhang	I agree to the proposed new authorship shown in section 4 /and the proposed change in corresponding author	Lei Zhang	5-Jan-2025
5th author	Jing	Wang	I agree to the proposed new authorship shown in section 4 /and the proposed change in corresponding author	Jing Wang	5-Jan-2025
6th author	Chunna	Xu	I agree to the proposed new authorship shown in section 4 /and the proposed change in corresponding author	Chunna Xu	5-Jan-2025
7th author	Lili	Dong	I agree to the proposed new authorship shown in section 4 /and the proposed change in corresponding author	LiLi Dong	5-Jan-2025

SPRINGER NATURE Springer Nature is one of the world's leading global research, educational and professional publishers, created in May 2015 through the combination of Nature Publishing Group, Palgrave Macmillan, Macmillan Education and Springer Science+Business Media.

扫描全能王 创建

SPRINGER NATURE

Change of authorship request form - Journals (pre-acceptance)

	First name	Family name		Signature	Date
8th author	Yanqiong	Wang	I agree to the proposed new authorship shown in section 4 /and the proposed change in corresponding author	Yanqiong Wang	5-Jan-2025
9th author	Yanyan	Su	I agree to the proposed new authorship shown in section 4 /and the proposed change in corresponding author	Yanyan Su	5-Jan-2025
10th author	Zhen	Dong	I agree to the proposed new authorship shown in section 4 /and the proposed change in corresponding author	Zhen Dong	5-Jan-2025
11th author	Haiming	Yang	I agree to the proposed new authorship shown in section 4 **/and the addition of my name to the authorship list** /and the proposed change in corresponding author	Haiming Yang	7-Jan-2025
12th author	Yuelin	Shen	I agree to the proposed new authorship shown in section 4 /and the proposed change in corresponding author	Yuelin Shen	7-Jan-2025

Please use an additional sheet if there are more than 10 authors.

SPRINGER NATURE Springer Nature is one of the world's leading global research, educational and professional publishers, created in May 2015 through the combination of Nature Publishing Group, Palgrave Macmillan, Macmillan Education and Springer Science+Business Media.

扫描全能王 创建

Change of authorship request form - Journals (pre-acceptance)

In case of author collaborations with formal agreement:

	Name of consortium/consortia	First name	Family name		Signature	Date
Representative/legal guarantor				I agree to the proposed new authorship shown in section 4 /**and the addition/removal*of my name to the authorship list** /and the proposed change in corresponding author		

Both added/removed authors should complete the information in the first table under Section 6.

---- End of form ----

SPRINGER NATURE Springer Nature is one of the world's leading global research, educational and professional publishers, created in May 2015 through the combination of Nature Publishing Group, Palgrave Macmillan, Macmillan Education and Springer Science+Business Media.
